# Untangling brain and behavioural measures of visual statistical learning: A longitudinal study in infancy

**DOI:** 10.1016/j.dcn.2026.101705

**Published:** 2026-03-09

**Authors:** Chiara Capparini, Lauréline Fourdin, Vincent Wens, Julie Bertels

**Affiliations:** a Laboratoire de Neuroanatomie et Neuroimagerie translationnelles (LN2T), ULB Neuroscience Institute (UNI), Université libre de Bruxelles (ULB), Brussels 1070, Belgium; b ULBabyLab – Center for Research in Cognition and Neurosciences (CRCN), ULB Neuroscience Institute (UNI), Université libre de Bruxelles (ULB), Brussels 1050, Belgium; c Department of Translational Neuroimaging, Hôpital Erasme, Hôpital Universitaire de Bruxelles (HUB), Université libre de Bruxelles (ULB), Brussels 1070, Belgium

**Keywords:** Statistical learning, Infancy, EEG, Frequency tagging, Neural entrainment

## Abstract

Statistical learning in infancy is typically studied using post-exposure behavioural paradigms, which can yield variable outcomes and pose interpretative challenges. Electrophysiological measures, recorded online during learning, offer a complementary approach. In this longitudinal study, we examined visual statistical learning in infants across three timepoints: 3 months (T1, *n* = 30), 6 months (T2, *n* = 27), and 9 months (T3, *n* = 23). At each session, infants participated in an EEG frequency-tagging paradigm involving 20-second sequences of shapes presented at 6 Hz, with deterministic doublets occurring at 3 Hz. Following familiarization, infants viewed familiar and novel doublets while looking times were recorded. Neural responses indicated sensitivity to the doublet structure in occipital regions as early as T1, with the learning index (entrainment at 3 Hz relative to 6 Hz) significantly increasing by T3. Behavioural looking times did not differ across ages, and a link between neural entrainment and behaviour was observed only in the older age group. These findings suggest that the infant brain can detect visual statistical regularities from early in life, with neural markers of learning becoming more robust and predictive of behaviour by 9 months. EEG provides a sensitive and continuous index of learning, capturing effects that may not always be evident in behaviour.

## 1 Introduction

From recognizing speech sounds to identifying visual cues, human infants can naturally detect patterns and regularities in their environment. This ability is called Statistical Learning (SL) and is most useful early in life (Saffran, 2020). Since seminal infant studies in the auditory (Saffran et al., 1996) and visual (Kirkham et al., 2002) domains, early SL skills have been typically assessed by measuring a behavioural outcome after a learning phase. Infants are commonly exposed to repeated sequences of stimuli organized in patterns until they become familiarized or habituated to them; at this point, novel sequences are presented to determine whether they can distinguish them from familiar ones presented earlier by means of difference in their looking behaviour. While a novelty effect is often taken as evidence of learning, the directionality of this novelty-familiarity preference varies according to individual and experimental factors (Bertels et al., 2022, Colombo and Bundy, 1983, Hunter and Ames, 1988, Roder et al., 2000), and also depending on the auditory vs. visual SL modality under investigation (Emberson et al., 2019). In turn, infants’ preference for novelty versus familiarity can be dynamic and may confound the interpretation of SL research (Houston-Price and Nakai, 2004). In this context, Batterink and Paller (2017) suggested that post-learning tests involve a memory-storage component that may be susceptible to individual differences in memory capacity. Further, it should be considered that similar post-exposure outcomes can emerge from different learning trajectories (Siegelman et al., 2018a). On top of that, neuroimaging studies have shown that adult learners exhibit neural markers of learning even in the absence of an explicit awareness, as indexed by above-chance behavioural recognition of regularities while participants claimed to respond randomly (Turk-Browne et al., 2009). Together, the above-mentioned findings support a shift in how SL can be evaluated - from relying solely on a single post-exposure behavioural outcome measure to also considering the online neural dynamics that unfold during learning itself.

One effective approach for capturing the online time course of learning is represented by neuroimaging techniques, especially electroencephalography (EEG) or magnetoencephalography (MEG), which have the temporal resolution needed to track online changes over the course of learning. Of note, the neuroimaging approach has the advantage to provide an online and dynamic measure of learning that is not affected by an overt behaviour. A first neuroimaging solution to address this is represented by Event Related Potentials (ERPs). For instance, an adult-like P300 component elicited by the visual statistics of the input pattern was found by the age of six (Jost et al., 2015). Even in infancy, violations of statistical regularities in action sequences elicited a Negative Central ERP response (Monroy et al., 2019). An alternative neuroimaging solution is to measure the online identification of regularities in the frequency domain, by means of frequency-tagged responses (also defined as steady-state evoked potentials or fast periodic stimulation). The frequency-tagging approach involves a rhythmic stimulation at a given frequency and measures neural responses evoked at the corresponding frequency. This is often associated with a broad definition of neural entrainment (e.g., Kabdebon et al., 2022). In the current work, we use the term entrainment to refer to the alignment of brain responses to the periodicity of external stimuli, irrespective of whether this alignment reflects true modulation of endogenous oscillations or a sequence of time-locked evoked responses. Notably, the frequency-tagging approach is especially well-suited for infants due to its robustness against artifacts and its suitability for short recording sessions (Kabdebon et al., 2022, Peykarjou, 2022).

In the field of SL, frequency-tagged brain responses can track the ongoing neural processes underlying the progressive detection of sensory regularities. So far, most SL studies investigated neural entrainment to auditory stimuli, in adults (e.g., Batterink and Paller, 2017, Batterink and Paller, 2019; Farthouat et al., 2017; Moser et al., 2021; Smalle et al., 2022) and even in infants (Choi et al., 2020, Kabdebon et al., 2015). Typically, this line of research includes the presentation of a continuous stream of syllables organised in trisyllabic pseudowords. The authors investigated entrainment to the embedded structure by means of peaks of neural activity at the word frequency or the ratio between the word and syllable frequency (the word learning index). Further, this approach has the capability to evaluate the evolution of neural entrainment over time. To this end, most auditory studies found an increased entrainment to the word presentation over time (Moreau et al., 2022, Moser et al., 2021, Kabdebon et al., 2015, Ordin et al., 2020, Smalle et al., 2022). While entrainment in auditory SL has been widely investigated due to its association with language acquisition, evidence of entrainment in the visual modality is more limited but still present (Henin et al., 2021, Sáringer et al., 2024). Notably, neural entrainment to visual regularities has been recently demonstrated even in 4- to 6-month-old infants (Capparini et al., 2026). In this previous work, infants were presented either with a doublet organization or with a random organization, and only those exposed to the doublet organization showed entrainment at the doublet frequency (Capparini et al., 2026). As opposed to auditory inputs, visual stimuli are particularly interesting in infancy as they are less influenced by native language interference and domain-specific expectations about linguistic structure (Siegelman et al., 2018b).

The possibility of obtaining an effective neuroimaging measure of the online dynamics of SL raises the question of whether online and post-exposure measures are linked, and whether neuroimaging can clarify and provide additional information on post-exposure behavioural findings. In infancy, this may have the potential to disambiguate unclear or unconclusive findings associated with the difficulty to predict the directionality of the behavioural learning outcome. Further, neuroimaging can also be more powerful to detect individual differences. In line with this, Saffran and Kirkham (2018) highlighted that a neural index seems a viable option to investigate individual differences that behavioural tasks cannot capture. Investigating the relationship between online neural entrainment to regularities and post-exposure behavioural learning outcome is a relatively new area of research. Some evidence even suggested that the manipulation of neural entrainment to regularities over the exposure phase can influence post-exposure learning outcomes and functionally contribute to SL (Batterink et al., 2024). Thus far, this relationship has been investigated in the language domain, especially in adults (see Sjuls et al., 2024 for a review). While half of the studies under review reported a positive association between neural and behavioural findings, the evidence seems still inconclusive at this stage (Sjuls et al., 2024). For instance, Moser et al., (2021) found that neural entrainment to auditory stimuli can predict subsequent explicit knowledge, even though the two processes were regarded as dissociable. Overall, reaction times in a target-detection task appear to be the measure most likely associated with neural entrainment in adult participants (Batterink and Paller, 2017, Batterink and Paller, 2019).

In infancy, investigations are limited and only two studies investigated this relation between online and post-learning tasks (Choi et al., 2020, Kabdebon et al., 2015). Both studies investigated the auditory modality and results are somehow contrasting. In Choi et al. (2020), 6-month-old infants showed a post-exposure novelty preference that was positively correlated with the online neural entrainment measured by the word learning index. According to the authors, increased neural entrainment to regularities reflects efficient learning and is later expressed as a preference for novel nonwords. At the same time, Kabdebon et al. (2015) did not find a link between entrainment to the word frequency during the exposure phase and the following learning outcome, this time measured by post-learning entrainment rather than a behavioural measure. In that work, neural entrainment to the syllable frequency rather than the word frequency was associated with the learning outcome in 8-month-old infants (Kabdebon et al., 2015). Crucially, to the best of our knowledge, the early emergence of this potential link during infancy has not been investigated in the visual domain. It is also possible that this link emerges at some point in development, a possibility that these two cross-sectional infant studies could not capture. Further research is needed, especially because the type of post-learning behavioural measure may play a role in whether a link between neural entrainment and behaviour can be found (Sjuls et al., 2024).

In the present work, we investigated infants’ neural entrainment to a continuous stream of shapes deterministically organized in doublets, alongside behavioural responses to novel vs. familiar doublets. This was investigated with a longitudinal design in 3-, 6-, and 9-month-old infants. In more detail, the procedure included a 2-minute familiarization phase, which assessed the time course of learning thanks to an EEG frequency-tagging approach, and a test phase, assessing post-exposure learning via looking times. This methodology allowed us to address two main aims. First, we examined whether neural entrainment to visual regularities is already present from 3 months of age and, if so, how entrainment values and the online learning trajectory evolve over the course of the first year. Second, we explored whether behavioural measures show evidence of learning and whether they relate to neural indices of learning over the course of infancy. In terms of neural entrainment results, we expected entrainment to the frequency of presentation of individual images (6 Hz) at all timepoints, reflecting simple visual perception of the images. We also expected entrainment to the doublet organization (3 Hz) at least from 6 months of age, based on a previous visual SL study using the same stimuli and a similar procedure, in which entrainment to doublets was observed only for structured sequences and not for random ones (Capparini et al., 2026). Overall, we expected to see a developmental trajectory of entrainment to the regularities, with relative sensitivity to the doublet organization increasing across timepoints. Regarding the online temporal dynamics of learning, if the regularities were detected, we expected high sensitivity to visual regularities from the initial sequences, in line with evidence showing that visual SL occurs rapidly and with little exposure (Capparini et al., 2026; Turk-Browne et al., 2009). Accordingly, entrainment to visual regularities may not need several sequences to emerge. At the same time, an increase in the learning index over timepoints was hypothesized. At the behavioural level, similar to Slone and Johnson’s behavioural paradigm (2018), the use of a familiarization phase rather than a habituation procedure did not allow us to predict the direction of preference (i.e., a novelty or familiarity effect) in the post-exposure test. Still, we expected a behavioural preference to become more marked with age. Lastly, as the limited evidence on a link between online and post-exposure measures of SL is inconclusive and only in the auditory domain (Choi et al., 2020, Kabdebon et al., 2015), we explored the link between neural and behavioural measures in the visual domain across timepoints. Given the rapid presentation rate used in this study to elicit frequency-tagged responses, which differs from previous behavioural SL paradigms, behavioural outcomes were treated as exploratory indicators rather than definitive evidence of brain-behaviour links.

## 2 Methods

### 2.1 Participants

A group of 30 3-month-old infants (*M*_*age*_ = 108.1 days, *SD* = 9.5 days; 17 females) was recruited for this longitudinal project including three timepoints (T1: 3 months, T2: 6 months, and T3: 9 months of age). All infant participants were born full-term (> 37 weeks), with normal birthweight (2.5 – 4.5 kg) and no complications at birth (Apgar scores > 7). Parents reported no sensory impairment nor developmental concerns at the time of testing. This was monitored via the Ages & Stages Questionnaires (ASQ-3; Squires and Bricker, 2009) administered at each timepoint. Infants lived in the surroundings of Brussels, Belgium. They were predominantly White (25 participants; 83.33%) and with Mixed racial background (4 participants; 13.33%); no data from one participant were available because of unknown paternal information. An additional 2 infants were recruited at 3 months of age but excluded from the final sample because of equipment error (*n* = 1) or incomplete data (*n* = 1); both did not come back for the following timepoints. A subgroup of 27 infants came back for T2 around 6 months of age (*M*_*age*_ = 194.1 days, *SD* = 9.8 days; 16 females). At T3, data were collected from a subgroup of 23 participants around 9 months of age (*M*_*age*_ = 288.6 days, *SD* = 8.8 days; 14 females). Hence, a total of 80 testing sessions were acquired across timepoints.

A caregiver provided informed written consent before the beginning of each testing session. The protocol of the study was approved by the Ethics Committee of the Hôpital Universitaire de Bruxelles (HUB), ref. P2019/480, B406201941700. The study was conducted according to the principles expressed in the Declaration of Helsinki.

### 2.2 Stimuli and apparatus

Stimuli included a set of eight colourful shapes on a uniform grey background displayed on a 30-inch LCD monitor (HP z30i, 1920 ×1200-pixel resolution, 60 Hz refresh rate). Each shape image was 800 × 800 pixels and subtended a visual angle of 13.5° at an 85 cm distance. Shapes were presented one at the time at the centre of the presentation screen with sinusoidal contrast modulation. A different set of eight colourful shapes was adopted at each timepoint to avoid any potential memory effect across timepoints (the type of set per timepoint was fixed across participants). An attention getting video with catching moving objects and sounds was used to attract the infant’s attention towards the screen before the start of each shape sequence.

Stimulus presentation was run in MATLAB (version R2017a, the MathWorks, Natick, MA, USA) with the Psychophysics Toolbox extensions (Brainard, 1997, Kleiner et al., 2007) on a computer controlled by the experimenter from a room adjacent to the testing room. Brain activity was recorded with a low-impedance, dense-array EEG system (Electrical Geodesic Inc., Eugene, Oregon, USA) at a sampling rate of 1000 Hz. The analog EEG signal was referenced to the vertex (Cz) and digitised by an EGI Net Amps 400 amplifier. EEG data were acquired with Net Station software, version 5.4.2 (r29917) running on a MacBook Pro Retina laptop computer. A set of 128-channel HydroCel Geodesic Sensor Nets covering different head perimeters was used to record the EEG signal. The LCD monitor in the testing room was equipped with an external video camera to monitor the participant’s behaviour during the experimental procedure. The video feed was recorded synchronously with EEG data on the acquisition computer.

### 2.3 Procedure

Infant participants sat on their caregiver’s lap at about 85 cm distance from the stimulation monitor in the testing room. At this point, infants were capped with the EEG net. Caregivers were instructed to hold their infant in a stable upright position in front of the monitor. They were also instructed to avoid speaking and interacting with their child during the experimental procedure. Lights were switched off and the testing room was only lit by the computer monitor to limit any potential distraction. EEG sensor impedance was checked just before the start of the experiment to ensure that all channels had an impedance below 50 kΩ.

**Fig. 1:**
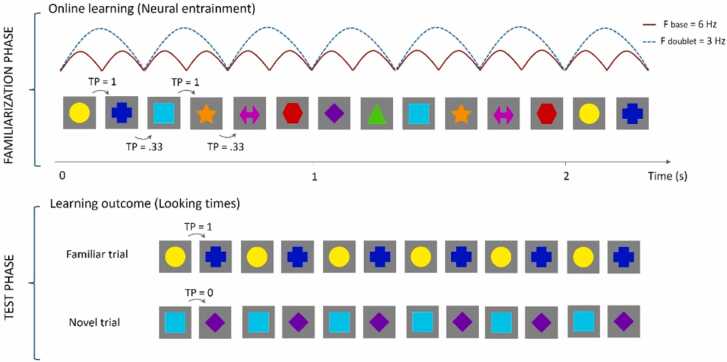
Schematic illustration of the experimental procedure, including the familiarization and test phases. The familiarization phase aimed to measure online learning mechanisms via EEG neural entrainment to visual regularities, while the test phase assessed offline post-exposure learning through looking times. Stimuli consisted of eight shapes presented with sinusoidal contrast modulation at a rate of six images per second (6 Hz; red line). During familiarization, shapes were presented in blocks of 24 s and organised into four doublets, yielding an intra-doublet transitional probability (TP) of 1 and an inter-doublet TP of 0.33. The doublet presentation frequency was three doublets per second (3 Hz; blue dotted line). The set of eight shapes changed at each timepoint. During the test phase, four familiar trials (repetition of a doublet already seen during familiarization, which had a TP of 1 during familiarization) and four novel trials (repetition of a shape pairing that was never presented during familiarization, hence with a TP of 0 during familiarization) were presented in alternation, each lasting 7 s.

The experimental procedure started with a familiarization phase, which was aimed at measuring the online temporal dynamics of learning via EEG frequency tagging (Fig. 1). This phase began with the presentation of the attention getting video. The experimenter controlled the presentation of this video and stopped it as soon as the participant was attentive, so that the following shape presentation could start. During familiarization, shapes appeared one at a time at a frequency of 6 Hz and were organized in blocks of 20 s. Fading periods of 2 s at the beginning and at the end of each block were used to reduce saccades and blinks caused by abrupt stimulation onset and offset. This led to extended blocks of 24 s with a total of 144 shapes, including the fading periods. Triggers were sent to the EEG system at each individual shape presentation, excluding the fading periods (i.e., for a total of 120 shapes per block). This continuous stream of eight shapes was organised in four doublets, so that each shape was followed by another shape in a deterministic organization (e.g., square always followed by star, circle always followed by triangle, cross always followed by arrow, rhombus always followed by hexagon). Hence, within doublets the likelihood of a shape occurring after another - that is, its transitional probability (TP) - was 1, whereas the TP between doublets was 0.33, with doublets pseudo-randomly ordered throughout the stimulation block (i.e., no consecutive presentation of the same doublet allowed). Shape pairing was randomised for each participant at the beginning of the procedure. The assigned shape pairing was maintained across blocks for the same participant. Of note, the present longitudinal design did not include a random control condition due to feasibility constraints associated with repeated infant testing. However, the specificity of the doublet entrainment effect at 3 Hz emerging only for structured sequences was established in a prior study using the same stimuli and EEG procedures, in which different groups of infants were exposed to deterministic doublets, non-deterministic doublets, and random sequences (Capparini et al., 2026). The familiarization phase lasted a minimum of 8 blocks, unless the procedure had to be interrupted prematurely (e.g., due to distress or fussiness). During the familiarization phase, the experimenter monitored the participant’s looking behaviour and recorded it online via a keypress. This allowed to check whether each participant obtained at least 120 s of cumulative looking after 8 blocks of familiarization. We used a 2-minute familiarization phase to ensure that all infants had a sufficient exposure to the visual regularities (this amount of time has been already used in similar infant paradigms investigating neural entrainment: Choi et al., 2020, Kabdebon et al., 2015) and also to make sure that infants had a comparable amount of time to encode the regularities at each timepoint. If this looking criterion was reached, participants were considered familiarized with the sequence and the familiarization phase was concluded, so that the test phase could begin. On the other hand, if the looking criterion was not reached at the end of the 8th block, an additional block was displayed until a total of 120 s of looking time had been accumulated (a maximum of 15 familiarization blocks were allowed to reach the looking criterion and proceed with the test phase; hence, if this limit was reached, only the familiarization phase data were available for analysis).

The following test phase, which was aimed at measuring post-exposure behavioural learning outcomes, included 8 trials. Two shapes were included in each test trial, presented one at the time at the centre of the screen in a continuous stream at 6 Hz, as it was the case during the earlier familiarization phase. These two shapes could be either a familiar doublet (i.e., a shape pairing already seen during the familiarization phase, whose TP during familiarization was 1) or a novel doublet (i.e., a shape pairing that was not presented earlier, hence with a TP of 0 during the familiarization phase). Out of eight test trials, four included a familiar doublet and four included a novel doublet. The presentation of familiar and novel trials was alternated. We chose to start always with a familiar trial as past research showed that SL is specifically associated with the arrangement of the initial test trials (Bertels et al., 2022). Each test trial lasted a total of 7 s, including 1 s fading periods at the beginning and end of the sequence. Hence, a total of 42 shapes were presented in each test trial. During this phase, the infant’s looking behaviour was measured offline based on video recordings. In total, the experimental SL procedure lasted between 5 and 10 min, depending on the participant’s looking behaviour and whether they could reach the test phase or not.

### 2.4 Data pre-processing and analysis

As first step, EEG data collected during the familiarization phase were pre-processed in MATLAB with the PREP pipeline to remove line noise, re-reference the signal relative to the average reference and automatically interpolate noisy channels (Bigdely-Shamlo et al., 2015). An average of 20.74 channels (*SD* = 10.13) out of 128 were interpolated per infant participant over a mean of 2.1 iterations. Events containing stimulation triggers were extracted to obtain segmented blocks of 20 s that did not include the fading periods. A Fast Fourier Transformation (FFT) was applied to the 20 s blocks, leading to a frequency resolution of 0.05 Hz. The strength of the response was quantified in terms of signal-to-noise ratio (SNR) between the power at the stimulated frequency and the estimated background noise. Specifically, the SNR was computed for each block as the ratio between the Fourier amplitude at the tagged frequency and the average amplitude of the 10 surrounding bins (5 bins on each side, excluding the two bins immediately adjacent to the tagged frequency; Peykarjou, 2022). At this point, valid blocks were selected to make sure that brain responses were not undermined by inattention. Selection of valid blocks included a two-step procedure, combining one electrophysiological step and one behavioural step, to make sure that visual stimuli were sufficiently attended. At the electrophysiological level, an SNR above 2 at the 6-Hz base frequency was required in at least one of ten occipital channels covering the medial occipital cortex (i.e., electrodes 69, 70, 73, 74, 75, 81, 82, 83, 88 and 89 of the 128-channel EGI net) that were a-priori defined and used in our previous infant study using a similar procedure (Capparini et al., 2026). Further, infants' behaviour was coded offline and blind from video recordings using ELAN software (version 6.4; Max Planck Institute for Psycholinguistics, Nijmegen, The Netherlands) to make sure that infants reached a looking-time criterion of at least 7 s per familiarization block (corresponding to approximately 42 shapes; as in Capparini et al., 2026). The coder had no information on what the participant was looking at. Blocks that passed both the electrophysiological and video-coding steps were considered valid. A minimum of two valid blocks per participant were required to be considered for further EEG analyses of the familiarization phase (Capparini et al., 2026).

Following valid block selection, tagged responses were analysed not only at the base frequency (6 Hz) and at the doublet frequency (3 Hz) but also at their harmonics (i.e., multiple integers of the frequency of interest). Of note, we considered as purely doublet frequency harmonics only those doublet harmonics not overlapping with base frequency harmonics (i.e., 9 Hz, 15 Hz, 21 Hz, etc.). Because the doublet frequency is mathematically related to the base frequency, some harmonic overlap is unavoidable, and the 6 Hz response may include contributions from the first harmonic of the doublets. Accordingly, the base-frequency response is interpreted as primarily reflecting stimulus-driven visual engagement, while sensitivity to statistical structure is solely captured at the doublet frequency and its non-overlapping harmonics. To determine how many harmonics to consider, we first visually inspected the mean SNR spectra to detect a frequency range of interest. Within this range, harmonic selection was further confirmed with one-sample Wilcoxon tests to ensure that mean SNR values at a given frequency were significantly greater than 1.

For the test phase, videos were coded offline with ELAN to estimate total looking times during each test trial. Every 100 ms, the coder judged if the infant was looking at the centre of the screen or looking away from it. The coder was not familiar with the experimental questions and unaware of what the participant was looking at. Total looking times per trial were then averaged across familiar and novel trials. A preference score was computed as the difference between average looking time at novel test trials and average looking time at familiar test trials. Hence, a positive preference score indicated an overall preference for novelty, whereas a negative score indicated a preference towards familiarity.

### 2.5 Statistics

Maximum statistics were performed on group-averaged whole-scalp SNR values, to localise electrodes with significant SNR at the base and doublet frequencies at each timepoint and, in turn, to determine regions of interest (ROIs) for the following analyses. For maximum statistics, a non-parametric permutation test was employed to generate surrogate data. Null distributions were based on 10000 permutations, testing the null hypothesis that the SNR was equal to 1. For each permutation, a random subset of subjects was selected, with each subject having a 50% probability of having their whole-scalp SNR topography replaced with a unit topography, while the remaining subjects retained their original SNR values. The group-averaged SNR was then computed, and the maximum across electrodes was recorded to build the permutation distribution. A significance threshold on SNR at significance level p < .05 was obtained as the 95th percentile of the permutation distribution of maximum value in group-averaged SNR topographies (Nichols and Holmes, 2002). The set of electrodes with SNR exceeding this threshold determined the ROIs at the group level, and individual SNR spectra were averaged across those electrodes. The resulting individual-level SNR values were finally averaged across selected harmonics, leading to one averaged value at the base frequency and another one at the doublet frequency per infant.

Linear mixed-effects models (LMMs) were adopted to model the evolution of SNR data across blocks and timepoints, using the lme4 package (Bates et al., 2015) implemented in R (R Core Team, 2023). This approach takes into account the unbalanced structure of the longitudinal dataset and models neural entrainment (measured by the SNR) at the block level. The models evaluated whether the fixed effects of block order and timepoint were related to the outcome variable, that is neural entrainment. Participant ID was included in all the models as a random factor to account for repeated measures. Since the SNR distribution is strictly positive, it was log-transformed prior to use in LMMs. Separate models were run to investigate entrainment at the base frequency, entrainment at the doublet frequency, and a doublet learning index (defined as the ratio between the average SNR at the doublet frequency and the average SNR at the base frequency per block, as in Choi et al., 2020). An increase of the learning index over time would reflect relatively stronger entrainment to the doublet frequency compared to the base stimulation frequency, which provides a normalized measure of structure-sensitive neural activity. Hence, this index captures the relative dominance of doublet-related responses over general stimulus-driven visual processing, rather than a pure measure of statistical learning independent of base-rate visual processing. Since our entrainment values were log-transformed, the learning index inputted to the LMMs was computed as the difference of log-transformed doublet-frequency SNR and log-transformed base-frequency SNR. Block order was computed following exclusion of invalid blocks (during which participants did not look sufficiently at the screen, as described in 2.4 Data pre-processing and analysis) and was renumbered so that the variable reflected the relative position of each valid block. Block order was truncated at 8 blocks since participants with more than 8 valid blocks were too sparse. Block order was also mean-centred prior to LMM analysis to facilitate interpretability of model coefficients, so that the model intercept represented the estimated brain response at the average block position. Further, to account for variability in participant attention across valid blocks (which could range from a minimum of 7 s to a maximum of 20 s of cumulative looking time per block), we included looking time as a covariate in the two LMM models investigating entrainment at the base frequency and entrainment at the doublet frequency. Looking time was also grand-mean centred prior to inclusion in the model, so that the intercept and main effects could be interpreted at average levels of attention. Behavioural data were also analysed with LMMs to test whether timepoint had an effect on the preference scores. Lastly, we examined whether there was a relationship between behavioural test data and neural familiarization data, and if this relationship differed across timepoints. This was evaluated by modelling the preference score as a function of the fixed effects of learning index at familiarization, timepoint, and their interaction. Confidence intervals (95% CI) for all LMM analyses were calculated using the Wald method. For all LMMs, we followed a stepwise modelling approach, progressively increasing model complexity by incrementally adding covariates (Hoffman and Rovine, 2007, Quené and Van den Bergh, 2004). We began with a parsimonious base model and incrementally added covariates of interest to examine their independent contributions to the outcome. Models were compared and selected by likelihood ratio test and changes in Akaike Information Criterion (AIC). The best-fitting models are reported in the Results section.

## 3 Results

### 3.1 Neural data (online learning)

#### 3.1.1 Valid blocks, data quality and harmonics selection

At 3 months of age, participants were exposed to an average of 7.97 familiarization blocks (*SD* = 2.55, range = 3–13 blocks, *n* = 239 blocks); 25 participants (83.33%) successfully familiarized with the shape sequence reaching the 2-minute cumulative looking criterion and 24 participants (80%) attended the test phase. At 6 months, an average of 8.77 familiarization blocks per participant (*SD* = 1.12, range = 8–11 blocks, *n* = 237 blocks) were recorded; all 27 participants reached the familiarization criterion and 24 (88.89%) attended the test phase. At 9 months, infants were exposed to an average of 8.43 blocks (*SD* = 1.41, range = 5–12 blocks, *n* = 194 blocks); 19 participants (82.61%) reached the familiarization criterion, and 17 participants (73.71%) attended the test phase.

Out of a total of 670 recorded blocks, 512 (76.42%) were considered valid and further analysed (see Methods, Data pre-processing and analysis, on valid block selection), leading to an average of 6.4 valid blocks of 120 images per participant. In more detail, 193 valid blocks were obtained at T1, 189 valid blocks at T2, and 130 valid blocks at T3. Of note, the average looking time per 24- s familiarization block did not vary across timepoints (M at T1 = 19.29 s; M at T2 = 18.95 s; M at T3 = 18.93 s). The decrease of blocks across timepoints is, at least in part, due to attrition of the longitudinal design (cumulative participants’ dropout rate of 23.33% from T1 to T3). Only one participant at T1 did not reach the minimum number of two valid familiarization blocks to be considered for further EEG analyses.

For harmonics selection, visual inspection of the mean SNR spectra revealed peaks in the 3 – 24 Hz range at all timepoints. Within this frequency range, the average base-level SNR at 6, 12, 18, and 24 Hz significantly exceeded 1 (all *p* < .001; Wilcoxon signed-ranks tests). At the doublet frequency, the average SNR at 3, 9, 15, and 21 Hz significantly exceeded 1 (all *p* < .001). These frequencies of interest were thus included in the following ROI analyses. Hence, when referring to the SNR at the base or doublet frequency in the LMMs analyses we considered the combined SNR obtained by averaging the above-described significant harmonics at the base (6, 12, 18, and 24 Hz) and doublet frequencies (3, 9, 15, 21 Hz), respectively.

#### 3.1.2 ROI analysis

**Fig. 2:**
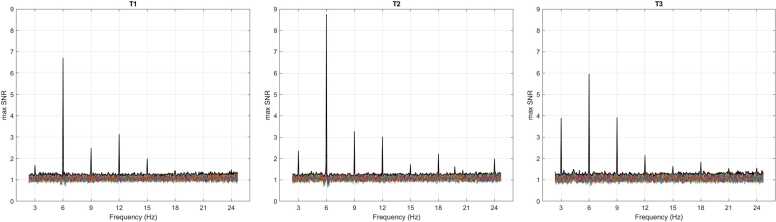
Signal-to-noise ratio (SNR) spectra across EEG channels at three timepoints (T1, T2, and T3). Each coloured line represents the average SNR across frequencies of interest for one EEG channel. The thick black line represents the maximum SNR across all channels at each frequency.

**Fig. 3:**
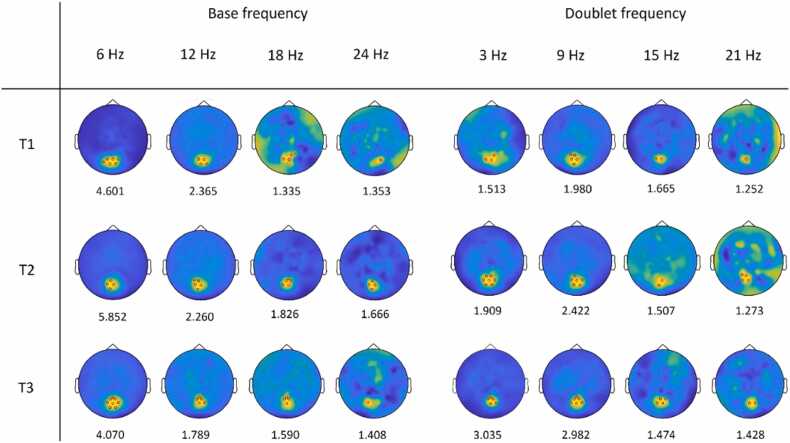
Topographical maps of brain activity at the base and doublet frequencies of interest across the three timepoints (T1, T2, and T3). For each frequency of interest, electrodes whose SNR values exceeded the significance threshold (*p* < .05) are highlighted with a red dot in each map. The SNR threshold for each frequency is reported below the corresponding map. Topographical maps are shown using the full colour scale for each frequency of interest, with yellow areas representing the highest SNR values and dark blue areas representing the lowest SNR values.

Spectra of SNR across timepoints are reported in Fig. 2. Maximum statistics revealed a cluster of electrodes with significant SNR that were consistently localized over the medial occipital area; that was the case across all timepoints and frequencies of interest (Fig. 3). No other electrodes reached significance outside the occipital area. Overall, at the base frequency and its harmonics, 6 Hz was the frequency with highest SNR and the largest number of occipital electrodes reaching significance at all timepoints. On the other hand, the frequency of 9 Hz showed the highest SNR threshold among the doublet-level frequencies. A cluster of 7 medial occipital electrodes (namely, 70, 71, 74, 75, 76, 82, 83) that showed significant SNR activity at both 3 Hz and 6 Hz across multiple timepoints was chosen as ROI for the following LMM analyses.

#### 3.1.3 Base stimulation responses (6 Hz and harmonics)

**Fig. 4:**
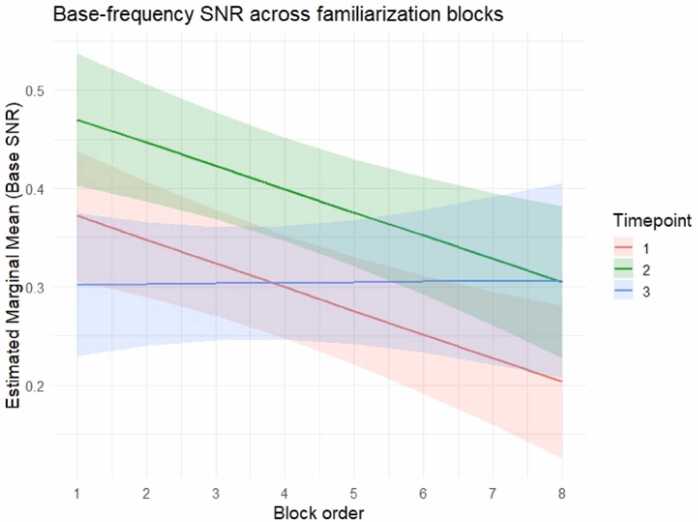
Estimated marginal means of base-level SNR (log-transformed) across familiarization blocks. Lines represent model-estimated marginal means for each timepoint (T1 in red, T2 in green, and T3 in blue) and shaded areas indicate 95% confidence intervals. Block order was entered as a centered continuous predictor in the model; values are displayed here as original block numbers (1−8) for interpretability.

At the group level, the base stimulation response showed a developmental peak at 6 months (T2) and a general decline over blocks, with this decline being attenuated at the latest developmental timepoint (Fig. 4). Specifically, the best-fitting model included fixed effects for Timepoint, Block order, their interaction, and Looking time as a continuous covariate (Table 1). The LMM revealed a significant main effect of Timepoint, with T2 showing increased base-level SNR compared to T1 (T2: *b* = 0.100, *SE* = 0.022, *t*(478) = 4.477, *p* < .001). No significant difference emerged at T3 relative to the baseline T1 (*p* = .936). Block order was also a significant predictor (*b* = −0.024, *SE* = 0.007, *t*(472) = –3.366, *p* < .001), suggesting an overall decline of the base-level SNR as familiarization progressed.

**Table 1:** The LMMs results of the base stimulation response (6 Hz and harmonics).

**Fixed Effects**							
	**Estimate**	**SE**		**95% CI**	**t value**		***p***
**Intercept**	0.302	0.026		0.251, 0.353	11.579		< .001 ***
**T2**	0.100	0.022		0.056, 0.143	4.477		< .001 ***
**T3**	0.002	0.025		-0.047, 0.051	0.082		.094
**Block Order**	-0.024	0.007		-0.038, −0.010	-3.366		.001 ***
**Looking Time**	0.029	0.003		0.023, 0.034	10.265		< .001 ***
**T2: Block Order**	0.001	0.010		-0.020, 0.020	0.046		.963
**T3: Block Order**	0.025	0.012		0.002, 0.048	2.119		.035 *
**Random Effects**							
	**Variance**		**SD**			**95% CI**	
**Intercept**	0.013		0.114			0.007, 0.024	
**Residual**	0.043		0.207			0.037, 0.048	

Significance codes: “***” p-value [0,.001] and “*” p-value [.01,.05]. Confidence intervals calculated using the Wald method. Model equation: Base-level SNR ∼ Timepoint * Block Order + Looking Time + (1 | Participant).

Crucially, these effects were qualified by a significant interaction between Block order and Timepoint (*b* = 0.025, *SE* = 0.012, *t*(469) = 2.119, *p* = .035), suggesting that the SNR decline over blocks differed across developmental stages, with a reduced decline at T3 (Fig. 4). Post hoc pairwise comparisons adjusted with the Holm method confirmed this pattern. Marginal means were estimated at three representative values of Block order that represent low, average, and high levels of the familiarization time course (-1 SD, 0, and +1 SD around mean, as in Preacher et al., 2006). At low block order, base-level SNR was significantly higher at T2 compared to both T1 (*b* = 0.099, *t*(472) = 3.123, *p* = .004) and T3 (*b* = 0.149, *t*(465) = 4.470, *p* < .001). Similarly, at average block order the SNR at T2 was significantly higher than T1 (*b* = 0.100, *t*(478) = 4.472, *p* < .001) and T3 (*b* = 0.098, *t*(472) = 3.967, *p* = .0002). At high block order, the base-level SNR at T2 remained significantly higher than T1 (*b* = 0.101, *t*(474) = 3.294, *p* = .003). However, differences between T2 and T3 (*b* = 0.046, *t*(472) = 1.256, *p* = .278) and between T1 and T3 (*b* = −0.055, *t*(475) = - 1.481, *p* = .278) were not statistically significant, reflecting a stabilization of the base response at this later timepoint. To assess the robustness of the interaction, the same comparisons were also conducted at −2 SD, 0, and + 2 SD around mean. The general pattern of results remained unchanged, confirming the stability of the interaction.

Finally, looking time was a strong positive predictor of base-level SNR (*b* = 0.485, *SE* = 0.047, *t*(488) = 10.265, *p* < .001), indicating higher neural responses during periods of greater attention. In this model, the intraclass correlation coefficient (ICC) was 0.23, indicating that approximately 23% of the variance in the base-level SNR was attributable to between-subject differences.

#### 3.1.4 Doublet individuation responses (3 Hz and harmonics)

**Fig. 5:**
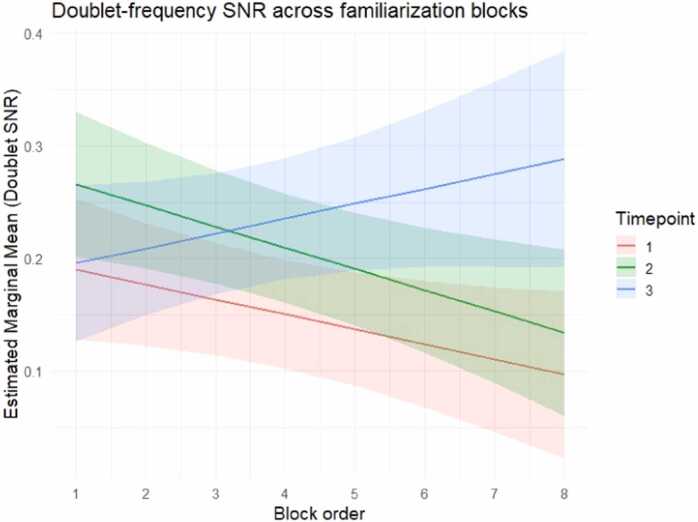
Estimated marginal means of doublet-level SNR (log-transformed) across familiarization blocks. Lines represent model-estimated marginal means for each timepoint (T1 in red, T2 in green, and T3 in blue) and shaded areas indicate 95% confidence intervals. Block order was modelled as a centered continuous predictor; values are displayed here as original block numbers (1−8) for interpretability.

At the group level, the doublet-level SNR showed a progressive increase over developmental stages, with stronger effects emerging at later blocks particularly at T3 (Fig. 5). More precisely, the best-fitting model for the doublet-level SNR included fixed effects of Timepoint, Block order, their interaction, and Looking time as a covariate (Table 2). The LMM revealed a significant doublet-level SNR increase at both T2 (*b* = 0.060, *t*(480) = 2.704, *p* = .007) and at T3 (*b* = 0.082, *t*(482) = 3.329, *p* = .001) relative to T1. The main effect of Block order approached significance (*b* = −0.013, *t*(474) = –1.878, *p* = .061), suggesting a possible decline over blocks.

**Table 2:** The LMMs results of the doublet individuation response (3 Hz and harmonics).

**Fixed Effects**							
	**Estimate**	**SE**		**95% CI**	**t value**		***p***
**Intercept**	0.152	0.024		0.105, 0.199	6.333		< .001 ***
**T2**	0.060	0.022		0.016, 0.103	2.704		.007 **
**T3**	0.083	0.025		0.034, 0.131	3.329		< .001 ***
**Block Order**	-0.013	0.007		-0.027, 0.001	-1.878		.061
**Looking Time**	0.018	0.003		0.012, 0.023	6.419		< .001 ***
**T2: Block Order**	-0.006	0.010		-0.025, 0.014	-0.541		.589
**T3: Block Order**	0.027	0.012		0.004, 0.049	2.293		.022 *
**Random Effects**							
	**Variance**		**SD**			**95% CI**	
**Intercept**	0.010		0.099			0.005, 0.018	
**Residual**	0.042		0.205			0.037, 0.048	

Significance codes: “***” p-value [0,.001], “**” p-value [.001,.01], and “*” p-value [.01,.05]. Confidence intervals calculated using the Wald method. Model equation: Doublet-level SNR ∼ Timepoint * Block Order + Looking Time + (1 | Participant).

Importantly, this pattern was qualified by a significant interaction between Timepoint and Block order (*b* = 0.027, *t*(471) = 2.293, *p* = .022), suggesting that the progression of the doublet-level SNR over familiarization blocks differed across timepoints, with a stronger late-emerging increase at T3 (Fig. 5). Post hoc analyses adjusted for multiple testing with the Holm method clarified this interaction, estimating marginal means at three levels of Block order (-1 SD, mean, and +1 SD). During early blocks, no reliable differences were observed across timepoints. At average block order, doublet-level responses were significantly higher at T2 (*b* = −0.060, *t*(479) = -2.699, *p* = .014) and T3 (*b* = −0.083, *t*(482) = -3.327, *p* = .003) relative to T1. At high block order, a pronounced SNR increase was observed at T3 relative to both T1 (*b* = −0.139, *t*(477) = -3.793, *p* < .001) and T2 (*b* = −0.091, *t*(474) = -2.519, *p* = .024), indicating that structure-related entrainment was strongest at the latest developmental timepoint and later familiarization stages. For robustness, the same comparisons were also conducted at −2 SD, 0, and + 2 SD around mean, and results interpretation remained unchanged, confirming the stability of the interaction.

Additionally, looking time was again a significant positive predictor (*b* = 0.018, *t*(490) = 6.419, *p* < .001), revealing higher doublet-level SNR during periods of greater attention. The ICC of this model was 0.19, suggesting that approximately 19% of the variance was attributable to between-subject differences.

#### 3.1.5 Doublet learning index

**Fig. 6:**
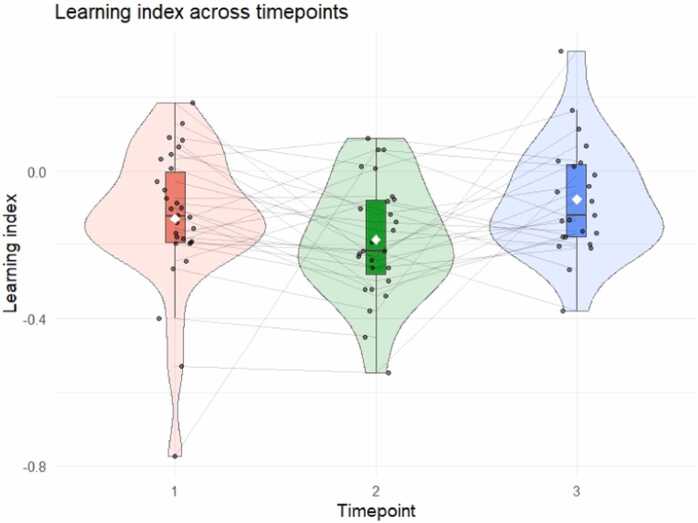
Learning index across three timepoints (T1, T2, and T3). The learning index was defined as the difference between log-transformed doublet-frequency SNR and log-transformed base-frequency SNR. Violin plots depict the full distribution of values; embedded boxplots indicate the median and interquartile range. Black jittered points represent individual participants, with lines connecting repeated measures across timepoints. The group mean learning index at each timepoint is represented by a white diamond. Higher values indicate stronger relative entrainment to the doublet frequency compared to the base frequency.

At the group level, the learning index showed a clear developmental increase, with the highest values at T3. In addition, it increased modestly but significantly as familiarization blocks progressed, indicating a gradual strengthening of structure-related entrainment over time. More specifically, the best-fitting LMM included fixed effects of Timepoint and Block order (Table 3). The model revealed a significant main effect of Timepoint, with T3 showing significantly higher learning index values relative to the baseline at T1 (*b* = 0.082, *SE* = 0.031, *t*(491) = 2.701, *p* = .007), whereas no difference was observed at T2 relative to T1 (*b* = - 0.037, *SE* = 0.027, *t*(488) = - 1.340, *p* = .181). Post hoc pairwise comparisons based on estimated marginal means (Tukey-corrected for multiple comparisons; Kenward-Roger degrees of freedom) confirmed a significant difference between T3 and T1 (*t*(491) = -2.694, *p* = .020), and between T3 and T2 (*t*(483) = -3.944, *p* < .001). The contrast between T1 and T2 was not significant, *t*(488) = 1.338, *p* = .375 (Fig. 6).

**Table 3:** The LMMs results of the learning index.

**Fixed Effects**								
	**Estimate**		**SE**		**95% CI**	**t value**		***p***
**Intercept**	-0.153		0.024		-0.201, −0.106	-6.298		< .001 ***
**T2**	-0.037		0.027		-0.090, 0.017	-1.340		.0181
**T3**	0.082		0.031		0.023, 0.142	2.701		.007 **
**Block Order**	0.012		0.006		0.001, 0.023	2.108		.0355 *
**Random Effects**								
		**Variance**		**SD**			**95% CI**	
**Intercept**		0.006		0.080			0.002, 0.014	
**Residual**		0.066		0.257			0.058, 0.075	

Significance codes: “***” p-value [0,.001], “**” p-value [.001,.01], and “*” p-value [.01,.05]. Confidence intervals calculated using the Wald method. Model equation: Learning index ∼ Timepoint + Block Order + (1 | Participant).

Block Order also had a small but significant positive effect on the learning index (*b* = 0.012, *SE* = 0.006, *t*(479) = 2.108, *p* = .036), indicating an overall increase of the learning index over time. Of note, the model fit was not improved by the inclusion of an interaction term between Block order and Timepoint and was therefore not retained, suggesting a similar temporal increase across timepoints. The ICC of the learning index model was approximately 0.09, suggesting that 9% of the variance was attributable to between-subject differences.

### 3.2 Behavioural data (post-exposure learning)

**Fig. 7:**
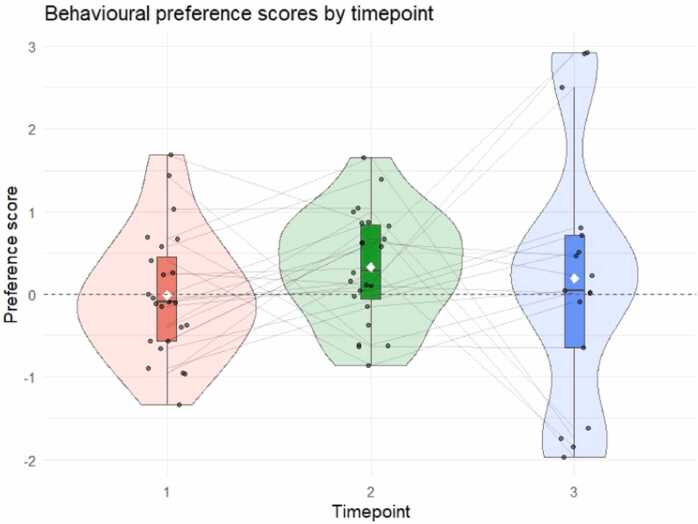
Distribution of preference scores across three timepoints. Mean preference score is defined as the difference between looking times towards novel trials and familiar trials. Each violin shows the distribution of scores at a given timepoint (T1 in red, T2 in green, and T3 in blue). Individual participant scores are represented by black dots (jittered for visibility) and connected by light lines to show within-participant trajectories. The white diamond indicates the group mean and error bars represent the standard error of the mean. The dashed horizontal line at 0 indicates no preference. Positive values indicate a preference for novel stimuli and negative values indicate a preference for familiar stimuli.

Overall, infants did not show a reliable preference for novel versus familiar doublets at any timepoint. Preference scores did not significantly differ from zero, with only a non-significant trend towards novelty preference at T2 (Fig. 7). Specifically, an LMM examining the effect of Timepoint on preference scores did not reveal any difference at T2 (*b* = 0.336, SE = 0.287, *t*(62) = 1.170, *p* = .247) nor T3 (*b* = 0.198, SE = 0.315, *t*(62) = 0.628, *p* = .532) relative to the baseline at T1. To ensure robustness, instead of collapsing to one aggregated preference score per subject, we also modelled trial-level looking times considering trial type (familiar, novel) and timepoint as fixed effects. Similarly, the model did not reveal any significant effect. These two model outcomes are reported as Supplementary Material (Tables S1 and S2).

To further explore whether participants showed a novelty preference at any timepoint, we conducted an exploratory follow-up analysis with one-sample *t*-tests comparing preference scores obtained at each timepoint to zero. Although an uncorrected analysis suggested a significant novelty preference at T2 (*t*(23) = 2.400, 95% CI [0.045, 0.608], *p* = .025), Holm-Bonferroni adjusted p-values indicated that none of the effects were statistically significant (Table 4). Preference scores did not significantly differ from zero, only a trend towards a novelty preference was observable at T2 (Fig. 7). Overall, these behavioural analyses suggest that no reliable or statistically robust novelty preference emerged over the first year of infancy.

**Table 4:** One-sample *t*-test results for preference scores at each timepoint. P-values were adjusted for multiple comparisons using the Holm-Bonferroni method.

**Timepoint**	**Mean Preference**	**SE**	***t***	**df**	**95% CI**	***p***
1	-0.009	0.155	-0.060	23	-0.331, 0.312	1.000
2	0.327	0.136	2.400	23	0.045, 0.608	0.074
3	0.189	0.373	0.506	16	-0.601, 0.979	1.000

### 3.3 Relation between neural and behavioural data

**Fig. 8:**
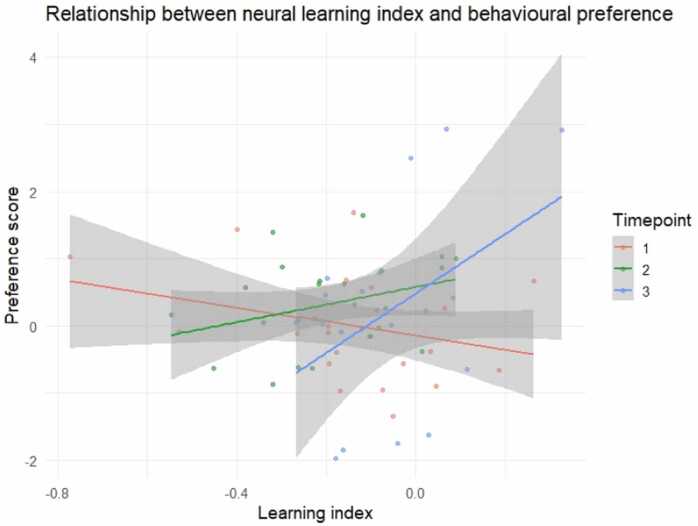
Relationship between neural learning index and behavioural preference score across timepoints. Each point represents a participant. Coloured lines show linear regression fits for each timepoint (T1 in red, T2 in green, and T3 in blue), with shaded areas indicating 95% confidence intervals. Results suggest that the relationship between learning and preference emerged over development, with higher neural learning index associated with a behavioural novelty preference score at T3.

The relationship between neural learning and behavioural preference strengthened over development. Specifically, at T3, infants with higher neural learning indices were more likely to exhibit a novelty preference, whereas no such association was observed at earlier timepoints (Fig. 8). To examine this relationship, we fitted a LMM predicting behavioural preference score from neural learning index and timepoint. The best-fitting model included fixed effects for Learning index, Timepoint, and their interaction. The model revealed a significant interaction between mean learning index and timepoint 3 (*b* = 5.468, *SE* = 1.809, *t*(59) = 3.022, *p* = .004), indicating that the association between neural entrainment and behavioural preference differed across developmental stages and was stronger at the final timepoint. Although the model included a random intercept for participant ID, the random-effect variance was estimated at zero, suggesting there was no meaningful between-subject variability in intercepts. This applied even running the model with subjects having data from at least two valid timepoints. Because fixed-effect estimates were virtually identical to those obtained from a standard linear model, we retained the linear model for this analysis, as it ensures more stable estimates and avoids overparameterization. This did not affect the interpretation of the results. The overall model was significant (*F*(5, 56) = 2.412, *p* = .0475), explaining about 17.7% of the variance (R² =.177, adjusted R² = 0.104). Again, the interaction between mean learning and T3 was significant, *b* = 5.390, *SE* = 1.810, *t* = 2.978, *p* = .004, indicating a stronger positive relationship at the final timepoint. The model outcome is reported in Table 5.

**Table 5:** Linear regression predicting the preference score from learning index, timepoint, and their interaction.

	**Estimate**	**SE**	**95% CI**	**t value**	***p***
**Intercept**	-0.070	0.231	-0.533, 0.394	-0.302	.764
**Learning index**	-0.969	0.899	-2.770, 0.832	-1.078	.286
**Timepoint 2**	0.762	0.392	-0.024, 1.548	1.942	.057
**Timepoint 3**	0.554	0.341	-0.130, 1.237	1.623	.110
**Learning index: T2**	2.641	1.535	-0.434, 5.716	1.721	.091
**Learning index: T3**	5.390	1.810	1.765, 9.016	2.978	.004 **

Significance code: “**” p-value [.001,.01]. Model equation: Preference score ∼ Learning Index * Timepoint + (1 | Participant).

To clarify the interaction, simple slopes were estimated to examine how the relationship between the mean neural learning index and behavioural preference varied across timepoints. At T3, a higher learning index was associated with stronger novelty preference (*b* = 4.421, *SE* = 1.571, 95% CI [1.270, 7.568], *p* = .007). In contrast, no significant association was observed at T1 (*b* = - 0.969, *SE* = 0.899, 95% CI [-2.770, 0.832], *p* = .286) or T2 (*b* = 1.672, *SE* = 1.244, 95% CI [-0.820, 4.164], *p* = .184). Pairwise comparisons adjusted with Tukey method revealed that the slope at T3 was significantly steeper than at T1 (difference = 5.390, *SE* = 1.810, *t*(56) = –2.978, *p* = .012), confirming that the link between neural sensitivity to statistical structure and behavioural novelty preference strengthens over the first year. No other pairwise slope differences reached significance (*ps* >.20, Tukey-adjusted).

## 4 Discussion

This longitudinal study investigated the development of neural and behavioural measures of visual SL across three timepoints in infancy (3, 6, and 9 months of age). On top of common post-exposure behavioural measures of learning (looking times to novel and familiar items), we focused on the time course of learning by assessing EEG neural entrainment during online exposure to visual regularities.

The first key finding is that SNR spectra revealed successful individuation of the rhythmic organization of the visual stimuli, with peaks at the doublet individuation frequency of 3 Hz and harmonics across developmental stages. This doublet individuation response was there already from 3 months of age, but it progressively increased with development, so that it was maximal at 9 months. This is in line with evidence of sensitivity to visual regularities already operational from 2 months of age (Kirkham et al., 2002). Brain topographic maps revealed a medial occipital activation at the doublet frequency and harmonics, which was similar in terms of brain localization across timepoints and frequencies of interest. In visual frequency-tagging paradigms, higher-order regularities may modulate activity within early visual processing networks rather than giving rise to clearly separable scalp distributions. Importantly, overlapping scalp distributions do not imply identical neural sources, since the limited spatial resolution of EEG constraints source separation. These results are in line with our recent study using a similar procedure in a group of 4- to 6-month-old infants (Capparini et al., 2026). In that study, neural entrainment at the doublet frequency was observed for structured sequences but not for random sequences, while the stimulation frequency response was present across sequence types. The present findings extend this evidence by showing robust neural entrainment in response to visual regularities from an even earlier age in a longitudinal design. Further, we showed that looking time played a role in doublet-level SNR, with a positive association between looking duration and doublet-frequency entrainment. In the auditory domain, attention does not always predict entrainment to higher-order regularities (Batterink and Paller, 2019), although Niesen et al. (2020) suggested that discrimination of words can be enhanced by top-down attention. An even clearer association with looking time may be expected to detect *visual* regularities, as infants’ attention directly determines exposure to the stimuli and entrainment is higher in blocks in which participants look more. While this may reflect top-down attentional modulation, it is important to note that longer looking also provides more usable EEG data, increasing the reliability and signal-to-noise ratio of frequency-tagged responses. This mechanical contribution may explain part of the observed association.

Importantly, maximal entrainment to visual regularities at 9 months of age was not explained by attentional mechanisms or differences in data quality alone. As a matter of fact, entrainment to the doublet organization was not strictly predicted by entrainment at the stimulation frequency, which is thought to index attention to the stimuli (Morgan et al., 1996, Toffanin et al., 2009). Indeed, entrainment to the stimulation frequency of 6 Hz and harmonics was present across all timepoints, with a peak at 6 months of age, and was strongly predicted by overt attention, measured as trial-level looking time towards the screen during familiarization. A combination of improvements in visual processing and oculomotor control by 6 months of age (e.g., Colombo, 2001; Johnson, 1990), may partly explain why this age group showed higher entrainment at the base frequency compared to younger infants. At the same time, 6-month-olds showed improved procedural compliance with the EEG procedure compared to older infants. Hence, the base-frequency SNR results may reflect an optimal engagement with the stimuli by the second timepoint. Despite these factors, doublet-frequency entrainment continued to increase and reached its maximum at 9 months, suggesting that the observed developmental increase reflects genuine sensitivity to visual regularities and the consolidation of statistical learning, beyond effects of data quantity or general attentional engagement.

Further, the temporal dynamics of entrainment to both the stimulation and doublet frequencies across blocks of exposure revealed intriguing findings. At the stimulation frequency, entrainment decreased as the exposure phase progressed. Interestingly, this neural repetition suppression was not evident at 9 months of age, timepoint which was characterized by a rather stable base-level entrainment over blocks of exposure. The base-level SNR decrease over time at earlier timepoints may also reflect fatigue or disengagement from the repeated visual stimulation, with a developmental transition around 9 months of age, where enhanced sustained attention can play a key role (Kannass et al., 2006). At the same age, different temporal dynamics also emerged at the doublet frequency when compared to those observed at earlier timepoints. Doublet-level entrainment reached its peak at 9 months during the last valid blocks of exposure, whereas it did not differ across timepoints at the initial stages of exposure. Importantly, entrainment to the regularities was already evident from the first valid blocks at all timepoints, consistent with evidence that detection of visual regularities can emerge with very little exposure (Capparini et al., 2026; Turk-Browne et al., 2009). A further developmental change that emerged at the last timepoint was the equally strong contribution of the frequencies of 3 Hz and 9 Hz to the doublet-level SNR spectrum, whereas at the first two timepoints the dominant doublet-level harmonic was 9 Hz. This shift in harmonic contribution may reflect a developmental refinement in the neural representation of structured input, with learning-related responses becoming distributed across multiple harmonics rather than concentrated at a single frequency. The different involvement of harmonics across timepoints confirms the importance of including multiple harmonics in the analyses, rather than focusing exclusively on the fundamental frequency (Peykarjou, 2022).

The decrease in entrainment to the regularities over time at T1 and T2 may have two contrasting interpretations. Either statistical regularities are so consolidated to become familiar and the infant is creating some predictions that reduce the processing demands, or the attentional and cognitive capacities are not sufficient to maintain engagement and fatigue emerges early on. While both interpretations are plausible, the developmental trajectory of the temporal evolution of doublet entrainment across timepoints and the different dynamics revealed at T3 - the only timepoint that showed an increase of doublet entrainment over blocks - speak in favour of the latter option.

Overall, we argue that the mere presence of entrainment at the doublet frequency, as revealed by average SNR spectra and topographic maps, reflects an early sensitivity to the statistical structure embedded in the streams of shapes (transitional probabilities), whereas changes in entrainment magnitude over exposure likely index the progressive consolidation of these regularities. In fact, entrainment to regularities was present at all timepoints and from the first valid blocks of exposure. However, the evolution of entrainment across blocks of familiarization revealed compelling differences across timepoints. Accordingly, it seems crucial to model time-resolved changes of doublet SNR over blocks to shed light on the temporal dynamics and strengthening of learning rather than relying solely on average SNR across blocks. In the present design, a block-level approach was adopted because reliable estimation of frequency-tagged responses requires sufficiently long and independent data segments to preserve frequency resolution and signal-to-noise ratio. Moreover, in infant visual paradigms, effective exposure depends on overt attention and may fluctuate within blocks, making finer-grained fixed-window segmentation difficult to interpret.

With this in mind, we further explored the temporal dynamics of learning by computing a learning index, which considered doublet-level entrainment relative to base-level entrainment at each block. The learning index was introduced as a normalization metric to account for individual and developmental differences in overall stimulus-locked responsiveness. It reflects relative dominance of structure-related entrainment over stimulus-driven entrainment within individuals. In the present study, the learning index was significantly higher at T3 compared to T1 and T2. Overall, it increased as blocks progressed. The model was not improved by a timepoint by block order interaction, suggesting that the increase over time was there at all timepoints. An increase of this index has been interpreted in previous speech processing work as reflecting a progressive detection of higher-order units relative to lower-level ones (Buiatti et al., 2009). Since then, several auditory studies reported the relative increase of entrainment towards words relative to syllables as an online index of SL (Batterink and Paller, 2017, Batterink and Paller, 2019, Choi et al., 2020). The present work extended the exploration of the neural dynamics of SL to the visual domain and investigated these developmental trajectories over the course of infancy for the first time. While the learning index generally increased at all developmental stages under investigation, the current data also highlighted how its time course was driven by different temporal dynamics at the base and doublet frequencies across timepoints. Specifically, at T1 and T2, both base-level and doublet-level SNR decreased over time, with a steeper decrease at the base frequency compared to the doublet frequency. Indeed, the repetition suppression phenomenon evidenced by entrainment at the stimulation frequency at T1 and T2, persisted in the entrainment at the doublet level, but to a lesser extent, which generated an enhanced learning index over blocks. By contrast, at T3 the learning index growth over time resulted from a relatively flat base-level SNR combined with a progressive increase in doublet-level SNR. In this context, the learning index is most informative when developmental or individual differences affect global signal amplitude or overall responsiveness, because it provides a relative measure of structure-sensitive activity normalized by stimulus-locked engagement. We therefore recommend using it as a complementary index alongside the doublet-frequency response, rather than as a standalone measure of statistical learning.

These neural findings raised the question of whether participants at all timepoints would show a learning outcome following familiarization, as suggested by an increase of the learning index at all ages, or whether a certain threshold and/or temporal dynamics of entrainment to regularities are necessary to show a post-exposure effect. Post-exposure behavioural data and their link with the neural findings during exposure were explored to shed light on the relationship between the online learning phase and the post-exposure learning outcome. First, behavioural results alone did not reveal any difference in looking time towards novel vs. familiar doublets across timepoints. In more detail, no preference was there at T1, a trend towards novelty was observable at T2 and results showed more extreme individual novelty or familiarity scores at T3, which overall did not reveal any preference. Albeit these null behavioural results, linking the post-exposure behavioural outcome with the earlier neural data during familiarization revealed an interesting relationship. Namely, a clear link emerged between the relative neural entrainment to doublets at 9 months and the post-exposure behavioural measure. In particular, the 9-month-olds with high neural learning index were the ones who showed a novelty preference at the behavioural test. Interestingly, a trend towards the same neural-behavioural association started to emerge at 6 months of age, even though it was not as marked as at 9 months. These results suggest that 9-month-old infants obtaining a higher neural learning index may be more efficient learners who, in turn, would be more likely to focus on the novel doublets compared to those doublets they had already learned. A similar link between behavioural preference score and learning index has been found in 6-month-old infants exposed to linguistic stimuli (Choi et al., 2020). Notably, the possibility of drawing some individual differences during the exposure phase via an EEG frequency-tagging approach allowed us to reveal learning outcomes in a subgroup of participants, which would have been hindered from group-level behavioural data alone.

Overall, the present results suggest that neural entrainment at the regularity frequency alone does not necessarily translate into a measurable behavioural learning outcome, particularly under conditions of rapid stimulus presentation. This suggests that the mere presence of frequency-specific responses should not be interpreted as unequivocal evidence of learning. Instead, examining the temporal dynamics of these neural responses across blocks of exposure appears critical to characterize learning and cautiously relate neural and behavioural measures of visual SL. Only at later stages of infancy an enhanced doublet SNR relative to the stimulation SNR was associated to a behavioural learning outcome. This raises the possibility that a sufficient doublet entrainment threshold should be reached to manifest learning in terms of behaviour. The fact that this link becomes clear at 9 months of age may be related to better retention and more efficient learning. For instance, it is only after 6 months that mismatch negativity responses to violations in statistical regularities become robust (Jing and Benasich, 2006). Alternatively, the behavioural measure may not be sensitive enough to capture the online learning mechanisms revealed during the exposure phase. In particular, during the test phase, familiar trials preserved the learned within-doublet transition but also introduced a reverse transition that was not experienced during familiarization, whereas novel trials consisted exclusively of unlearned transitions. This graded difference may have reduced the sensitivity of the behavioural measure, especially in younger infants. Likewise, the stimulus presentation rate of 6 Hz, which is standard in frequency-tagging paradigms with infants (e.g., Bertels et al., 2020; Kabdebon et al., 2022; Peykarjou, 2022), may have been too challenging to elicit robust differences in terms of behaviour. While this option does not undermine the current neural findings nor the observed link between stronger neural entrainment and novelty behavioural preference in older infants, it may raise the possibility that the behavioural task was too demanding for the youngest group of participants. Indeed, previous behavioural studies revealing a novelty/familiarity preference adopted a slower presentation frequency, usually around 1 Hz (e.g., Bertels et al., 2022; Tummeltshammer et al., 2017). In the current study, keeping the stimulation frequency constant was considered the best option to ensure consistency between the familiarization and test phases and to avoid introducing a degree of temporal novelty into the familiar doublet presentations by slowing down the frequency of presentation at test. Given the rapid presentation rate used in this paradigm, behavioural outcomes were considered exploratory and neural entrainment provided the primary and most sensitive index of learning. Future studies may investigate if slower presentation rates are beneficial for younger infants and may lead to clearer behavioural outcomes and stronger links with neural data even at earlier developmental stages. More generally, post-exposure behavioural preference alone may not be well-suited to capture individual differences or the different pathways and temporal trajectories that lead to learning. These considerations further support the relevance of focusing on the temporal dynamics of neural responses to regularities revealed during exposure, in order to better understand how successful learning unfolds over time.

To conclude, tracking the dynamics of learning over development revealed important insights into visual processing of regularities. The present work suggests that behavioural post-exposure scores alone may provide only a partial view of individual differences and developmental changes unfolding during the exposure phase. In this context, a combination of neural online tracking and post-exposure behavioural outcomes may offer a more informative approach for characterizing learning mechanisms and their developmental trajectories.

## CRediT authorship contribution statement

**Chiara Capparini:** Writing – review & editing, Writing – original draft, Visualization, Validation, Software, Project administration, Methodology, Investigation, Formal analysis, Data curation, Conceptualization. **Lauréline Fourdin:** Writing – review & editing, Investigation. **Vincent Wens:** Writing – review & editing, Supervision, Software, Formal analysis. **Julie Bertels:** Writing – review & editing, Supervision, Resources, Project administration, Methodology, Funding acquisition, Conceptualization.

## Funding

This work was supported by the FRS-FNRS incentive grant for scientific research, number F.4503.22. Further funding was provided by the Fondation-JED Belgique and by the Fondation Jaumotte-Demoulin.

## Declaration of Competing Interest

The authors declare that they have no known competing financial interests or personal relationships that could have appeared to influence the work reported in this paper.

## Data Availability

Data will be made available on request.

## Footnotes

Appendix A Supplementary data associated with this article can be found in the online version at doi:10.1016/j.dcn.2026.101705.
