# Supplementary material for: Untangling brain and behavioural measures of visual statistical learning: A longitudinal study in infancy
Source: Dev Cogn Neurosci. 2026 Mar 9;79:101705. doi: 10.1016/j.dcn.2026.101705 (PMC13054608; doi:10.1016/j.dcn.2026.101705)
Supplement: Supplementary file 1 — Supplementary material [file mmc1.docx]

## Supplementary Materials

| Fixed Effects | | | | | | | |
| --- | --- | --- | --- | --- | --- | --- | --- |
|  | **Estimate** | **SE** | **95% CI** | | **t value** | | ***p*** |
| Intercept | -0.009 | 0.203 | | -0.407, 0.389 | -0.046 | | .936 |
| T2 | 0.336 | 0.287 | -0.227, 0.899 | | 1.170 | | .247 |
| T3 | 0.198 | 0.315 | -0.420, 0.816 | | 0.628 | | .532 |
| Random Effects | | | | | | | |
|  | **Variance** | **SD** | | | | **95% CI** | |
| Intercept | 0.000 | 0.000 | | | | 0.000, 0.204 | |
| Residual | 0.989 | 0.995 | | | | 0.682, 1.359 | |

## **Table S1**. Results from the LMM model exploring the effect of timepoint on the behavioural preference score. Confidence intervals calculated using the Wald method. Model equation: Preference score ~ Timepoint + (1 | Participant).

| Fixed Effects | | | | | | | | |
| --- | --- | --- | --- | --- | --- | --- | --- | --- |
|  | **Estimate** | **SE** | | **95% CI** | | **t value** | | ***p*** |
| Intercept | 4.234 | | 0.144 | | 3.953, 4.516 | 29.504 | | <.001 *** |
| Trial Type (novel) | 0.186 | | 0.142 | | -0.092, 0.463 | 1.312 | | .190 |
| T2 | 0.208 | | 0.164 | | -0.114, 0.530 | 1.265 | | .206 |
| T3 | 0.121 | | 0.187 | | -0.246, 0.488 | 0.647 | | .518 |
| Random Effects | | | | | | | | |
|  | **Variance** | | **SD** | | | | **95% CI** | |
| Intercept | 0.060 | | 0.245 | | | | 0.000, 0.207 | |
| Residual | 2.466 | | 1.571 | | | | 2.162, 2.795 | |

## **Table S2**. Results from the LMM model exploring the effect of trial type (novel; familiar) and timepoint on looking times during the test phase. Significance codes: “***” p-value [0, .001]. Confidence intervals calculated using the Wald method. Model equation: Looking time ~ Trial Type + Timepoint + (1 | Participant).
